# Aerostability of Sin Nombre Virus Aerosol Related to Near-Field Transmission

**DOI:** 10.3390/pathogens14080750

**Published:** 2025-07-30

**Authors:** Elizabeth A. Klug, Danielle N. Rivera, Vicki L. Herrera, Ashley R. Ravnholdt, Daniel N. Ackerman, Yangsheng Yu, Chunyan Ye, Steven B. Bradfute, St. Patrick Reid, Joshua L. Santarpia

**Affiliations:** 1 Department of Pathology, Microbiology, and Immunology, University of Nebraska Medical Center (UNMC), Omaha, NE 68198, USA; liz.klug@unmc.edu (E.A.K.); vlherrera@unmc.edu (V.L.H.); aravnholdt@unmc.edu (A.R.R.); dackerman@unmc.edu (D.N.A.); yangshengyu@unmc.edu (Y.Y.); patrick.reid@unmc.edu (S.P.R.); 2 The Global Center for Health Security, University of Nebraska Medical Center (UNMC), Omaha, NE 68198, USA; danielle.rivera@unmc.edu; 3 National Strategic Research Institute (NSRI), Omaha, NE 68198, USA; 4 Center for Global Health, Department of Internal Medicine, University of New Mexico, Albuquerque, NM 87131, USA; cye@salud.unm.edu (C.Y.); sbradfute@salud.unm.edu (S.B.B.)

**Keywords:** bioaerosol, Sin Nombre virus (SNV), decay, hantavirus cardiopulmonary syndrome (HCPS), Bio-ARC, flow-through system

## Abstract

Sin Nombre virus (SNV) is the main causative agent of hantavirus cardiopulmonary syndrome (HCPS) in North America. SNV is transmitted via environmental biological aerosols (bioaerosols) produced by infected deer mice (*Peromyscus maniculatus*). It is similar to other viruses that have environmental transmission routes rather than a person-to-person transmission route, such as avian influenza (e.g., H5N1) and Lassa fever. Despite the lack of person-to-person transmission, these viruses cause a significant public health and economic burden. However, due to the lack of targeted pharmaceutical preventatives and therapeutics, the recommended approach to prevent SNV infections is to avoid locations that have a combination of low foot traffic, receive minimal natural sunlight, and where *P. maniculatus* may be found nesting. Consequently, gaining insight into the SNV bioaerosol decay profile is fundamental to the prevention of SNV infections. The Biological Aerosol Reaction Chamber (Bio-ARC) is a flow-through system designed to rapidly expose bioaerosols to environmental conditions (ozone, simulated solar radiation (SSR), humidity, and other gas phase species at stable temperatures) and determine the sensitivity of those particles to simulated ambient conditions. Using this system, we examined the bioaerosol stability of SNV. The virus was found to be susceptible to both simulated solar radiation and ozone under the tested conditions. Comparisons of decay between the virus aerosolized in residual media and in a mouse bedding matrix showed similar results. This study indicates that SNV aerosol particles are susceptible to inactivation by solar radiation and ozone, both of which could be implemented as effective control measures to prevent disease in locations where SNV is endemic.

## 1. Introduction

Sin Nombre virus (SNV) is the main causative agent of hantavirus cardiopulmonary syndrome (HCPS) in North America. Early symptoms of HCPS are nonspecific and include fatigue, fever, and muscle pain. Later symptoms involve respiratory distress that requires immediate medical attention and has a 30% fatality rate [1]. SNV is an enveloped, negative-sense, single-stranded RNA virus belonging to the *Hantaviridae* family and *Bunyavirales* order. First isolated in 1993 following an outbreak in the four corners region of the United States, it has continued to cause infections and mortality, primarily in North America [2]. SNV is transmitted via contaminated environmental biological aerosols (bioaerosols) produced by infected deer mice (*Peromyscus maniculatus*) [3,4]. This differs from other enveloped viruses that transmit via person-to-person contact, such as SARS-CoV-2. However, this transmission route is similar to other viruses that have an environmental transmission route, such as avian influenza (e.g., H5N1) and Lassa virus. These viruses lack a person-to-person transmission route; however, they have a large impact on agricultural economics, human morbidity and mortality, and the economy [5,6,7,8,9]. This has been especially prevalent with the 2025 outbreak of H5N1 and the culling of different bird species, such as domestic chickens and turkeys [6]. Despite understanding SNV aerosol transmission dynamics, an SNV bioaerosol decay profile has not been established. This can make effective prevention difficult to successfully implement.

Humans are not the preferred host for SNV and are referenced as a “dead end host” because person-to-person transmission has not been observed [10]. Interestingly, a similar hantavirus, Andes virus (ANDV), has documented person-to-person transmission [11]. Furthermore, both SNV and ANDV have similar aerosol transmission pathways involving rodents, and the pathogen potentially enters human lung endothelial cells via human β3 integrins [12]. Unfortunately, the mechanism of viral entry is not well understood, but it is believed to be via endocytic vesicles and acidification of the host endosome [6]. Understanding this difference in transmission dynamics is a multidisciplinary discussion which requires an understanding of virology, immunology, and the physical and biochemical properties that impact this virus. Our aim with this study is to further this conversation by investigating the decay of SNV in the aerosol phase.

In this study, we used the Biological Aerosol Reaction Chamber (Bio-ARC) to study the early-stage bioaerosol decay phase of SNV [13]. This flow-through system exposes biological particles to controlled conditions for short periods of time. This allows the Bio-ARC to rapidly decay biological particles in a uniform way, allowing larger quantities to be collected for downstream analysis [13]. In this study, SNV bioaerosols were exposed to a variety of conditions, including simulated solar radiation (SSR) and ozone under midrange (~48.5%) relative humidity (RH) conditions.

Conditions for this study in the Bio-ARC were chosen to simulate environmentally relevant conditions that SNV bioaerosols can encounter in various real-world settings, including both indoor and outdoor environments. SSR was chosen based on the impact of UVA and UVB on bioaerosols [14]. Ozone was specifically chosen because it is a common component of both urban indoor and outdoor air that has been shown to impact bioaerosol stability [15,16]. Additionally, ozone can be used to decontaminate a space against bacterial and viral diseases, which could be useful for SNV prevention strategies [17,18].

## 2. Materials and Methods

### 2.1. Overview of Biological Aerosol Reaction Chamber (Bio-ARC)

The Bio-ARC is a system designed to rapidly expose biological aerosols to simulated environmental conditions and determine the sensitivity of those particles to simulated ambient conditions. A detailed description of the Bio-ARC system used for the comparison decay studies with the bacteriophage MS2 has been previously described in Klug et al. (2025) [13]. Further updates have been introduced to the Bio-ARC system to allow for streamlined BSL-3 performance. These include the addition of remote-controlled RH and temperature probes (S-THC-M008, Onset Computer Corporation, Bourne, MA, USA, and H21-USB, Onset Computer Corporation, USA). Remote-controlled flow controllers and meters (BB9-232, Alicat Scientific, Tucson, AZ, USA, and MCW-1SLPM-D and MCPW-2SLPM-D, Alicat Scientific, Tucson, AZ, USA) were added to the Bio-ARC to further streamline RH control.

**Figure 1:**
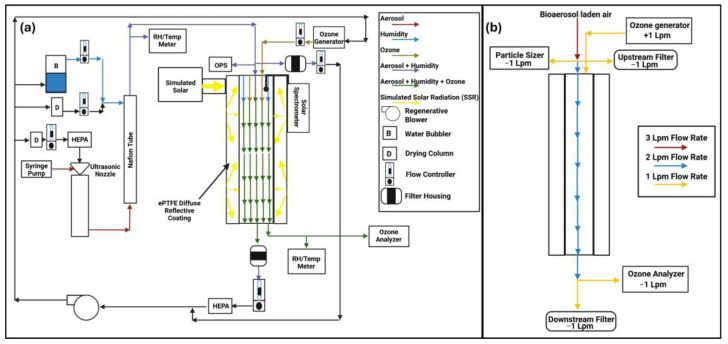
The Biological Aerosol Reaction Chamber (Bio-ARC). (**a**) The Bio-ARC schematic: The Bio-ARC is a system designed to rapidly expose biological aerosols to environmental conditions and determine the sensitivity of those particles to simulated ambient conditions. (**b**) Bio-ARC flow rates within the main exposure chamber. The Bio-ARC schematic was created with Biorender. Source: Klug, E. (2025), https://BioRender.com/g08q9bi. The Bio-ARC flow rate diagram was created with Biorender. Source: Klug, E. (2025), https://BioRender.com/wotq9dp.

A more significant modification was the addition of an upstream filter (Figure 1). The two-filter system allows every experimental replicate to have an internal control, eliminating the need for tracer materials, such as sodium fluorescein. The filter added before the main exposure chamber acts as a control for the secondary filter, which is placed at the end of the main exposure chamber. In this arrangement, unexposed bioaerosols that have been conditioned to experimental RH are collected in the upstream filter, whereas the second, downstream filter captures bioaerosols that have been exposed to the experimental conditions at the same RH, allowing for a more direct measurement of the impact of the chosen conditions on a replicate-by-replicate basis.

### 2.2. Bio-ARC Experimental Overview

Each experiment lasted ten minutes. Prior to each test, the system was preconditioned to the desired RH value and, if included, the desired simulated solar intensity or ozone concentration. A gelatin filter (12602-47-ALK, Sartorius Inc., Göttingen, Germany) was loaded into each filter housing prior to testing. Suspensions were aerosolized via a 120 kHz ultrasonic nozzle (06-04-00552-006, SONOTEK, Milton, NY, USA) with 3 lpm of carrier air (Figure 1b). The use of 3 lpm of carrier air is necessary to facilitate size distribution measurements (Optical Particle Sizer (OPS) (3330, TSI, Shoreview, MN, USA) at 1 Lpm) and upstream filter collection (1 Lpm) prior to introduction into the exposure chamber. Similar to Klug et al., the bioaerosols had a conditioning time of 45 s before impaction onto the upstream filter [13]. After the size distribution measurement and upstream filter collection, aerosol-laden air (1 Lpm) was co-introduced into the reaction chamber with 1 Lpm of ozone-laden or clean air. This 2 Lpm flow results in an exposure time of one minute in the 2 L reaction chamber prior to collection on the downstream gelatin filter and sampling by the ozone monitor (1 Lpm) (UV-100, ECO Sensors Inc., Santa Fe, NM, USA). Following exposure, both gelatin filters were collected and processed as described below to determine biological activity. Additionally, SNV suspensions used in the aerosolization syringe were tested to determine biological activity.

### 2.3. Experimental Conditions in Bio-ARC

The SNV decay profile was examined over midrange (~50%) RH conditions. Two SSR conditions were tested: lamp off and lamp at maximum output. As described in Klug et al. [13], at maximum output, the xenon arc lamp (6271, Newport Corp, Irvine, CA, USA), shaped by an air mass filter (81094, Newport Corp, Irvine, CA, USA) as measured by a spectrometer (BLK-C-SR, StellarNet, Tampa, FL, USA), results in SSR exposures of no UVC (100–280 nm), 0.01 $\frac{W}{m^{2}}\cdot hr$ of UVB (280–315 nm), 2.46 $\frac{W}{m^{2}}\cdot hr$ of UVA, and between 8.33 and 12.59 $\frac{W}{m^{2}}\cdot hr$ in the range of 400 to 1000 nm. Two target ozone conditions were used: 0 ppm (ozone generator off) and 1 ppm. Due to fluctuations in the output of the ozone generator and the 2% accuracy of the ozone monitor, a margin of ±10% was considered acceptable for the ozone target concentration. Temperature was not directly controlled in these experiments, but it was monitored throughout the experiments.

### 2.4. Cells

Vero cells (CCL-81, ATCC, Manassas, VA, USA) were cultured at 37 °C and under 5% CO_2_ in DMEM (11965-092, Thermo Fisher Scientific Inc. Gibco, Waltham, MA, USA) supplemented with 10.0% *v*/*v* heat-inactivated fetal bovine serum (FBS) (97068-085, Avantor Inc., Radnor Township, PA, USA) and 1% *v*/*v* antibiotic–antimycotic solution (15240-062, Thermo Fisher Scientific Inc. Gibco, Waltham, MA, USA).

### 2.5. SNV Propagation and Titration/Viability Assays

The SNV strain 77734 and anti-SNV rabbit sera were generously provided by Dr. Stephen Bradfute’s laboratory at the University of New Mexico (UNM). SNV propagation in VeroE6 cells followed methods reported in [19,20]. For this study, a T-75 flask of VeroE6 cells was infected with SNV at an MOI of 0.1. The infection lasted a total of 18 days with EMEM (10-009-CV, Corning Inc., Corning, NY, USA) supplemented with 2.5% *v*/*v* heat-inactivated FBS and 1% antibiotic–antimycotic collected and replaced every 3 days. SNV was stored at −80 °C until aerosolization.

SNV viability and titration were determined via a focus reduction neutralization test (FRNT). The FRNT was performed in VeroE6 cells, similar to the methods reported in [19,20]. For this study, serially diluted filter or matrix samples were added to VeroE6 cells plated at 1 × 10^4^ cells/well. These were incubated for 2 h at 37 °C. After incubation, an overlay containing 1.2% methylcellulose and 2× EMEM (11935-046, Thermo Fisher Scientific Inc. Gibco, Waltham, MA, USA) supplemented with 2.5% *v*/*v* heat-inactivated FBS and 1% *v*/*v* antibiotic–antimycotic solution was added to each well. The overlay was carefully removed after 7 days, and the cells were fixed with methanol. The addition of rabbit anti-SNV nucleocapsid protein serum was used to visualize the SNV antigen and was followed with peroxidase-conjugated goat anti-rabbit IgG and DAB/metal concentrate as substrates (Pierce) (34065, Thermo Scientific Inc., Waltham, MA, USA).

### 2.6. Titration of Collected SNV Bioaerosols

Filters were removed from the Bio-ARC and dissolved in 5.0 mL EMEM supplemented with 5.0% *v*/*v* heat-inactivated FBS and 1% *v*/*v* antibiotic–antimycotic solution. The filters were then incubated at 37 °C to allow the gelatin filter to dissolve. The infectious concentration of SNV samples was determined, in triplicate, via the FRNT, as described in Section 2.5.

### 2.7. SNV RT-PCR

RT-qPCR was used to calculate the estimated focus forming unit (eFFU/mL); this was performed to look at the possible impact of the simulated environmental conditions on SNV nucleic acids.

Gelatin filters were processed as mentioned above, with a 0.4 mL aliquot used for RT-qPCR analysis. To recover viral RNA, 0.4 mL of each recovered and dissolved filter sample was processed using a Qiagen EZ1&2™ Virus Mini Kit V2.0 (955134, QIAGEN, Hilden, Germany) on a Qiagen EZ1 Advanced XL (9001874, QIAGEN, Hilden, Germany). A negative extraction control with no sample added was included with each set of extractions. Samples were eluted in 60 μL of Qiagen AVE Buffer. RT-PCR was performed using the Invitrogen Superscript III Platinum One-Step Quantitative RT-PCR System (11732088, Thermo Fisher Scientific Inc., Waltham, MA, USA). Each RT-PCR run included a positive SNV control and a negative control, no template, and a control of nuclease-free water.

PCR cycling conditions included 10 min at 55 °C for cDNA synthesis and reverse transcription, 4 min at 94 °C, and 45 cycles at 94 °C for 15 seconds and 58 °C for 30 s, on a QuantStudio™ 3 (A28567, Applied Biosystems™ Inc., Foster City, CA, USA). The primers and probe were based on Botten et al. [20] and targeted the SNV S gene. Each sample was tested in triplicate.

### 2.8. Generation of SNV Matrices and Aerosol Particles

The SNV-relevant matrix was created using uninfected, soiled mouse bedding collected from uninfected mouse cages in the UNMC Comparative Medicine department. The mouse bedding consisted of corncob bedding (77097, Inotiv Inc., Lafayette, IN, USA), and the mice were fed a fixed mouse/rat sterilizable diet (7012, Inotiv Inc., Lafayette, IN, USA). Deionized water was added to soiled mouse bedding, and the solution was incubated at room temperature for 72 h. Following incubation, the liquid was decanted from the soiled mouse bedding and filtered using a 0.22 µm membrane (SCGP00525, MilliporeSigma, Burlington, MA, USA) to remove larger particles and bacteria. The following solution was used as the mouse bedding aerosolization matrix.

Two different matrices were used during these experiments. A spent cell culture media solution containing SNV propagated in EMEM with 2.5% FBS diluted in deionized water was employed. The mouse bedding aerosolization matrix contained a 1:10 dilution of SNV and the mouse bedding aerosolization matrix. The size of SNV aerosol particles generated in these experiments was characterized via an OPS. All aerosol matrices were injected via a syringe pump (11-01-00250, SONO-TEK, Milton, NY, USA) at 0.1 mL/min into the 120 kHz Sonotek nozzle to produce particles.

### 2.9. Data Analysis

#### 2.9.1. Statistical Analysis

Throughout these studies, statistical calculations to determine the significance of the findings were performed using GraphPad Prism version 10.4.1. The specifics of individual tests are given as they are described.

#### 2.9.2. Bio-ARC Decay Rate Calculations

To assess the viability of each collected sample, we performed an FRNT on downstream exposed filters and normalized that value using the FRNT results for the upstream exposed filter. The downstream filter was multiplied by 2 to correct for dilution occurring between the upstream and downstream filters when the particle-free airstream used to carry ozone into the exposure chamber (described in detail in Section 2.2 and in Figure 1b) is mixed with the bioaerosol-laden air. Additionally, the individual upstream and downstream filters from each run were compared against one another to understand if the environmental conditions had an impact (Supplemental Table S1). The following equation was used to calculate decay due to RH, SSR, or ozone exposure:

$$\frac{{log}_{10}\left(U_{FFU}\right)-{(log}_{10}\left(D_{FFU}\right)\;•\;2)}{t}$$

where:

*FFU*: SNV aerosol concentration (determined by FRNT FFU/mL).
*U*: upstream filter.
*D*: downstream filter, multiplied by two to correct for the air flow.
*t*: time from upstream to downstream sample filter.

## 3. Results

### 3.1. Bio-ARC System Characterization

**Figure 2:**
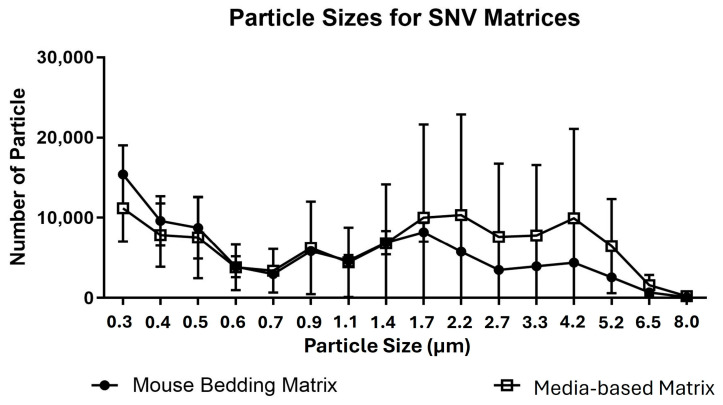
Particle characterization of biological aerosols produced within the Bio-ARC. This was performed with a TSI Optical Particle Sizer (OPS). Analysis was completed using GraphPad Prism.

The Bio-ARC system conditions were measured throughout the experimental trials. The overall RH was kept at a stable midrange RH target during the study at 48.5 ± 5.0%. The temperature was stable during all trials at 29.1 ± 10.8 °C. The target ozone concentration during this study was 1.0 ppm, with a measured mean of 1.1 ± 0.03 ppm. Measured spectral irradiance and total exposure varied by 2.94% over all SSR experiments. Carbon dioxide concentrations were measured in a previous study at 652.5 ± 30.0 ppm [13]. The FRNT was performed on the solution used for aerosolization to ensure that SNV viability did not decrease during Bio-ARC testing. One stock of SNV was used for the study, with the initial infectivity measured at 7.6 • 10^5^ ± 1.6 • 10^3^ FFU/mL. Particle characterization was performed with a TSI OPS; the results indicated a bimodal distribution for both the mouse bedding matrix and SNV media matrix with a peak at under a micron in size and a second peak under two microns (Figure 2).

### 3.2. Bio-ARC: SNV Bioaerosol Decay Rates

**Figure 3:**
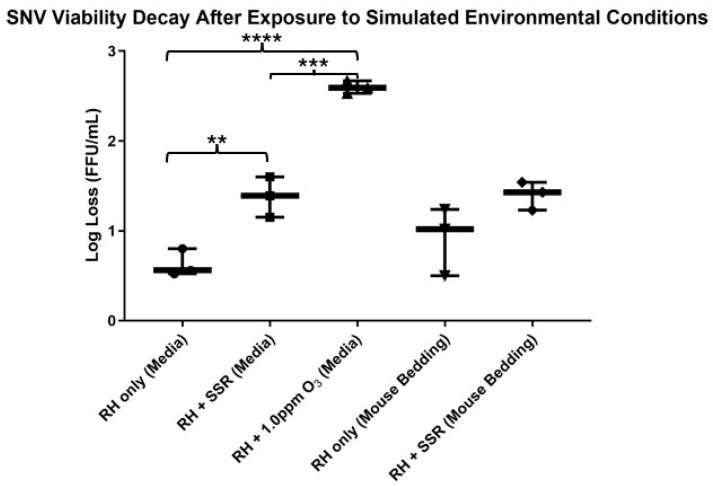
SNV decay rates witnessed within the Bio-ARC after exposure to a variety of simulated environmental conditions with two different matrices. SNV decay measured via the FRNT. An unpaired, two-tailed *t*-test was used to identify whether a statistically significant difference was found between SNV viabilities after exposure to different environmental conditions. Significance was determined if the *p* value was less than 0.05. The box and whisker plot displays the minimum and maximum bounds. ** indicates a *p* value ≤ 0.01; *** indicates a *p* value ≤ 0.001; **** indicates a *p* value ≤ 0.0001. An ordinary one-way ANOVA was also completed against all datasets with a *p* value < 0.0001, indicating significance. Analysis was completed using GraphPad Prism.

SNV decay rates were calculated considering the RH-only upstream filter as a negative control condition. Each experiment was run in triplicate. Based on an unpaired *t*-test, each replicate run from every condition had a significant difference between the upstream and downstream filter viabilities (Supplemental Table S1). When the baseline of only midrange RH (42.2 ± 1.3%) was investigated, the log decay rate of SNV was 0.6 ± 0.2 log/min (Figure 3). The log decay rate was increased with the addition of SSR at 1.3 ± 0.3 log/min at an RH of 40.5 ± 1.9% (Figure 3). At 49.1 ± 0.8% RH, the addition of 1.0 ppm ozone caused a significant increase in the amount of SNV decay at 2.6 ± 0.1 log/min (Figure 3). This is especially evident based on an unpaired *t*-test against RH-only decay rates. An ordinary one-way ANOVA was also used to confirm whether significant differences were present across all group means, regardless of condition or matrix. This returned a *p* value of less than 0.0001, indicating significance.

Two matrices were used in this study. In these experiments, SNV particles produced in a spent cell culture media solution (0.6 ± 0.2 log/min) decayed similarly compared to the more physiologically relevant mouse bedding aerosolization matrix (0.9 ± 0.4 log/min) at RH values of 42.2 ± 1.3% and 50.2 ± 0.4%, respectively (Figure 3). Similarly, the two matrices did not impact decay with the addition of SSR, where SNV in spent cell culture media decayed at 1.3 ± 0.3 log/min at an RH of 40.5 ± 1.9%, and SNV in mouse bedding matrix decayed at 1.49 ± 0.1 log/min at 51.1 ± 2.8%. There was no statistically significant difference in pathogen viability between the two matrices during decay at midrange RH with or without simulated sunlight.

### 3.3. Bio-ARC: SNV Bioaerosol RT-PCR

**Figure 4:**
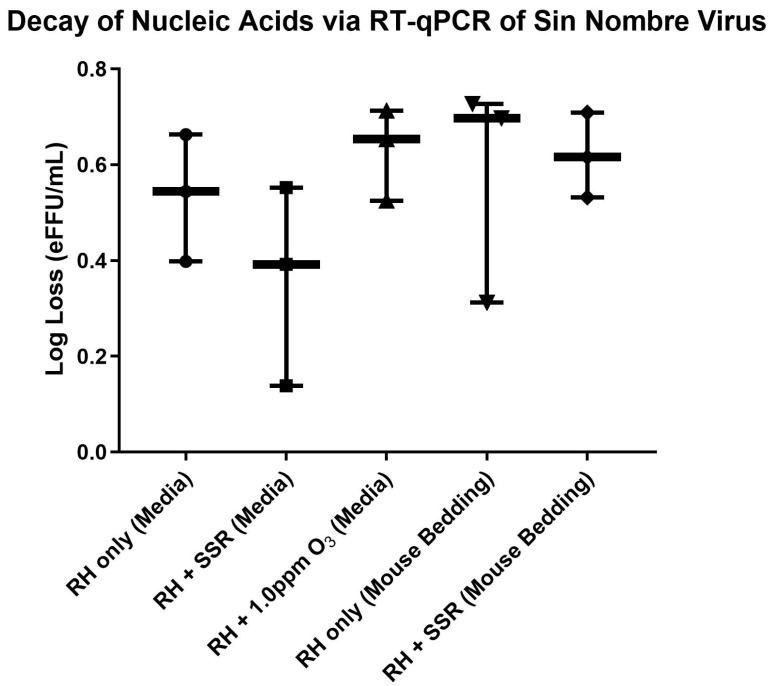
RT-qPCR results of SNV estimated log loss decay. An unpaired, two-tailed *t*-test was used to identify whether a statistically significant difference was found between the SNV estimated viabilities after exposure to different environmental conditions. The box and whisker plot displays the minimum and maximum bounds. No significance was measured based on a *p* value > 0.05. Analysis was completed using GraphPad Prism.

RT-PCR was used to elucidate whether SNV nucleic acids were being damaged during exposure to the simulated environmental conditions, and if this damage resulted in a loss of pathogen viability. SNV estimated decay rates were calculated considering the RH-only upstream filter as a negative control condition. Each PCR experiment was run in triplicate. Under only midrange RH conditions (48.5%), the estimated log decay rate of SNV was 0.5 ± 0.1 eFFU/min (Figure 4). The addition of secondary environmental conditions did not cause a statistically significant difference in the estimated log decay rate. The addition of SSR resulted in an estimated log decay of SNV at 0.4 ± 0.3 eFFU/min and the addition of 1.0 ppm ozone was 0.6 ± 0.1 eFFU/min. Based on these data, there is no indication that damage to nucleic acids, affecting this RT-PCR assay, is responsible for the observed loss of pathogen viability.

Additionally, the mouse bedding aerosolization matrix did not seem to confer additional protection to SNV nucleic acids. The estimated log loss of SNV infectivity (0.6 ± 0.2 eFFU/min) was not significantly different from either the spent cell culture media matrix or the addition of a secondary environmental condition. This is evident with the addition of SSR, with an estimated loss of SNV viability at 0.6 ± 0.1 eFFU/min (Figure 4).

## 4. Discussion

SNV was exposed to a variety of simulated environmental conditions within the Bio-ARC system to examine environmental conditions that impact the initial decay of SNV aerosols. The results from the Bio-ARC indicate that the impact of SSR on SNV decay was higher compared to decay under fixed RH conditions. This was expected due to the impact of UVA and UVB radiation. UVB radiation causes direct absorption damage; this causes free radical formation via electron oxidation or hydrogen atom abstraction [14]. UVA radiation is responsible for photosensitization damage causing the formation of singlet oxygen [14]. UV irradiation can also cause pyrimidine dimers, which can impact nucleic acids, RNA replication, and pathogen replication [21,22].

The results from the Bio-ARC indicate that ozone had the greatest impact on SNV viability and was statistically significant compared to midrange RH alone and SSR. Ozone is a highly reactive oxidant and causes ozonolysis, which allows carbon–carbon double bonds to be replaced by double bonds with oxygen [23]. Furthermore, hydrolysis of the primary ozonide causes peptide bond cleavage, protein–protein crosslinking, the oxidation of polypeptide backbones, and amino acid side-chain modifications resulting in pathogen inactivation [24,25]. These impacts are particularly damaging for major aromatic amino acids which contain carbon–carbon double bonds [23]. This may be an important factor when SNV is exposed to ozone. This is because the SNV Gn and Gc glycoproteins contain 1.3% of the aromatic amino acid, tryptophan [26].

Our data indicate that aerosolized SNV decays quickly at midrange (48.5%) RH. The addition of ozone and SSR increased the measured decay rate and indicates that aerosolized SNV is especially vulnerable to these environmental conditions. These findings offer insight into why SNV is primarily contracted in dark, isolated areas such as unused attics, barns, or sheds. Our results could indicate that human SNV infections could be reduced with the introduction of ozone or sunlight into a potentially SNV-contaminated space. A different, more physiologically relevant matrix was also used to compare with the laboratory-based approach of using spent cell culture media. The results indicate that there is not a statistically significant difference between the spent cell culture media matrix and the mouse bedding aerosolization matrix. This suggests that the differences between these two matrices did not impact the stability of aerosolized SNV under midrange RH conditions with or without the addition of SSR. However, it should be recognized that SNV aerosolized in the mouse bedding matrix did have a higher mean log loss and standard deviation than SNV aerosolized in the cell culture matrix. This shift, although not significant, results in a lack of significance with the addition of SSR when compared with the mouse bedding conditions.

To further the conversation of understanding what factor could be causing this loss in viability, RT-PCR was used to understand if SNV nucleic acids were being damaged during exposure to simulated environmental conditions. Based on our data, there is no indication that damage to nucleic acids is a primary factor causing the observed loss in SNV viability. Regardless of the environmental condition added or the matrix employed, the estimated loss of SNV viability did not change compared to the baseline condition. This study indicates that the loss of pathogen viability is more likely related to the degradation of viral lipids or proteins.

To aid in prevention strategies, the SNV bioaerosol decay profile needs to be explored and understood. Our research has begun to investigate the dynamic phase of SNV bioaerosol decay when exposed to a variety of simulated conditions, with and without a relevant aerosolization matrix, and physiologically relevant particle sizes [27,28]. Research into the severe acute respiratory syndrome coronavirus 2 (SARS-CoV-2) bioaerosol decay profile indicates a triphasic decay profile consisting of lag, dynamic, and slow decay phases [28]. The Bio-ARC targets measurements of decay during the dynamic decay phase in a way that allows for more detailed analysis of the aerosols by enabling large volumes of particles that have identical aging profiles to be collected and analyzed. This is distinct from other technologies, such as rotating drums, which look at decay primarily in the lag phase, or particle levitation systems, which use small particle numbers to map the entire profile. The advantages and disadvantages of each aerosol exposure system have been previously discussed at length in Santarpia et al. (2020) and Klug et al. (2025) [13,29]. Additional work to better characterize the entire decay profile would provide additional benefit, but the work described here addresses decay on the timescale most relevant to short-range transmission and typical environmental exposure events. Additionally, investigation into different hantavirus strains, such as ANDV, and different, more physiologically relevant matrices could be useful as well.

## 5. Conclusions

In conclusion, we have observed that simulated environmental factors cause a loss of SNV viability. Specifically, aerosolized SNV is especially susceptible to the introduction of sunlight and ozone within a space. Additionally, neither SNV nucleic acids nor the aerosolization matrix appear to drive the loss of viability. Instead, damage to proteins or lipids is the likely driver of decay under these conditions. More research is required to fully elucidate these mechanisms and reveal how they might apply to other conditions and pathogens.

There are various ways this study could be improved. An important note is the lack of *P. maniculatus* urine/excreta as a matrix for SNV. Our study was limited to healthy, lab-adapted mice bedding and did not include the intricacies that wild *P. maniculatus* urine/excreta would naturally contain. Furthermore, more simulated environmental conditions could be explored, like the impacts of ozone on SNV aerosolized in a mouse bedding matrix. However, we were limited to using the FRNT to understand SNV viability. It is important to mention that, due to a lack of the specific commercially available primary antibody required for focus forming assays, it is increasingly difficult to study SNV. Additionally, the temperature did fluctuate by ±10 °C during these experiments, which may have contributed to the observed variability in some conditions.

SNV causes the serious, potentially lethal condition HCPS. There is no available, specific treatment for HCPS; the most beneficial way to prevent HCPS is to avoid being in contact with SNV-infected rodents and their urine or excreta. For this reason, understanding the impact that environmental factors have on SNV bioaerosols will aid in developing prevention strategies. This is the first study to report the decay of SNV bioaerosols. The focus of this work on early-stage dynamic decay is critical to informing near-field transmission and prevention strategies. Further understanding of the SNV bioaerosol decay profile, namely the slow decay phase, is needed to fully understand decay as a whole. However, based on the epidemiology of SNV infections and the rapid decay of SNV under these conditions, it seems that the dynamic phase of bioaerosol decay is key to preventing SNV exposure and disease. Application of these findings could aid in the prevention of SNV infections. They demonstrate that exposure to simulated sunlight or treatment with ozone inactivates SNV and may help to prevent infection.

## Data Availability

The data presented in this study are openly available in the Harvard Dataverse at https://doi.org/10.7910/DVN/DMLNIB.

## Supplementary Materials

The following supporting information can be downloaded at https://www.mdpi.com/article/10.3390/pathogens14080750/s1.

## Abbreviations

The following abbreviations are used in this manuscript:

SNV	Sin Nombre virus
HCPS	hantavirus cardiopulmonary syndrome
Bioaerosol	biological aerosol
ANDV	Andes virus
Bio-ARC	Biological Aerosol Reaction Chamber
SSR	simulated solar radiation
RH	relative humidity
OPS	Optical Particle Sizer
FRNT	focus reduction neutralization test
eFFU	estimated focus forming unit
FFU	focus forming unit
